# Gymnodimine A and 13-desMethyl Spirolide C Alter Intracellular Calcium Levels via Acetylcholine Receptors

**DOI:** 10.3390/toxins12120751

**Published:** 2020-11-27

**Authors:** Joyce A. Nieva, Bernd Krock, Urban Tillmann, Jan Tebben, Christian Zurhelle, Ulf Bickmeyer

**Affiliations:** Alfred Wegener Institute, Helmholtz Center for Polar and Marine Research, D-27570 Bremerhaven, Germany; joyce.nieva@awi.de (J.A.N.); Bernd.Krock@awi.de (B.K.); urban.tillmann@awi.de (U.T.); jan.tebben@awi.de (J.T.); christian.zurhelle@awi.de (C.Z.)

**Keywords:** cyclic imine, nicotinic, muscarinic, and acetylcholine receptors, calcium signaling, *Alexandrium ostenfeldii*

## Abstract

Gymnodimines and spirolides are cyclic imine phycotoxins and known antagonists of nicotinic acetylcholine receptors (nAChRs). We investigated the effect of gymnodimine A (GYM A) and 13-desmethyl spirolide C (SPX 1) from *Alexandrium ostenfeldii* on rat pheochromocytoma (PC12) cells by monitoring intracellular calcium levels ([Ca]_i_). Using whole cells, the presence of 0.5 µM of GYM A or SPX 1 induced an increase in [Ca]_i_ mediated by acetylcholine receptors (AChRs) and inhibited further activation of AChRs by acetylcholine (ACh). To differentiate the effects of GYM A or SPX 1, the toxins were applied to cells with pharmacologically isolated nAChRs and muscarinic AChRs (mAChRs) as mediated by the addition of atropine and tubocurarine, respectively. GYM A and SPX 1 activated nAChRs and inhibited the further activation of nAChRs by ACh, indicating that both toxins mimicked the activity of ACh. Regarding mAChRs, a differential response was observed between the two toxins. Only GYM A activated mAChRs, resulting in elevated [Ca]_i_, but both toxins prevented a subsequent activation by ACh. The absence of the triketal ring system in GYM A may provide the basis for a selective activation of mAChRs. GYM A and SPX 1 induced no changes in [Ca]_i_ when nAChRs and mAChRs were inhibited simultaneously, indicating that both toxins target AChRs.

## 1. Introduction

The frequency and widespread occurrence of marine biotoxins associated with microalgae has increased over the years [1]. Among the emerging classes of lipophilic marine toxins are the macrocyclic imine compounds such as prorocentrolides, spiro-prorocentrimine, gymnodimines, spirolides, pinnatoxins, and portimine [2].

Spirolides, one of the classes of cyclic imine, are produced by *Alexandrium ostenfeldii* [3]. To this day, they have not been found in any other microalgal species. Gymnodimines, on the other hand, have only been recently reported to co-occur with spirolides in at least a number of *Alexandrium ostenfeldii* strains [4,5,6,7,8] and were initially identified in *Karenia selliformis* [9]. The presence of structurally related gymnodimines and spirolides in a single microalgal species suggests that both toxins share a common biosynthetic pathway. In addition to the cyclic imine moiety, the butenolide side chain is identical for gymnodimines and spirolides, indicating a common function in both toxin types [10].

**Figure 1:**
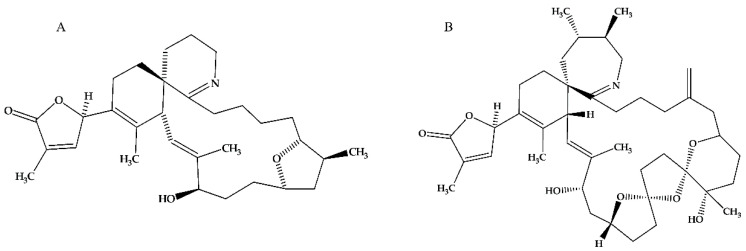
Chemical structure of Gymnodimine A (GYM A) (**A**) and 13-desmethyl spirolide C (SPX 1) (**B**) [16].

Functional bioassays on gymnodimine A (GYM A, Figure 1A) and 13-desmethyl spirolide C (SPX 1, Figure 1B) revealed a similar bioactivity. Since both induce rapid neurotoxic symptoms in mice after intraperitoneal injection or oral administration, they are called “fast-acting toxins” [11]. Moreover, results from in vivo assays showed that the time for symptoms to manifest shortened and the onset of death accelerated when cholinergic or acetylcholinesterase inhibitors were simultaneously administered. These observations prompted studies to determine the mechanism by which the toxins affect acetylcholine receptors (AChRs) [12,13]. Electrophysiological measurements using clonal cells demonstrated that while GYM A as well as SPX 1 broadly targets muscular and neuronal nicotinic AChRs (nAChRs) [14,15,16,17], only GYM A showed a reversible effect [14]. These studies used both homomeric and heteromeric subtypes such as α7, α1_2_β1γδ, α7-5HT_3_, α3β2, and α4β2 nAChRs. The effect of SPX 1 on muscarinic AChRs (mAChRs) was determined using a human neuroblastoma cell model. It showed the antagonistic effect of the toxin that resulted in a reduced function and decreased specificity of mAChRs [18]. However, recent studies contradicted this model and showed that neither SPX 1 [15,17] nor GYM A [17] strongly interact with mAChR subtypes M_1_–M_5_.

Only a few bioactivity studies other than the mouse bioassay [11] and in vitro inhibition assays [12,13,14,15,16,17,18] have been performed on gymnodimines and spirolides. Electrophysiological measurements were limited to a specific receptor, which was overexpressed [19]. Considering that GYM A and SPX 1 have shown anticholinergic effects on nAChRs in recombinant cells [12,13,14,15,16,17,18], it is important to determine the effects on cellular signaling.

In neuroendocrine cells such as rat pheochromocytoma (PC 12) cells, many different types of receptors and ion channels are simultaneously present [20,21]. Voltage-gated calcium (Ca) channels and AChRs are known to permeate calcium ions (Ca^2+^) into the cell when the channels are opened as a result of depolarization or binding of acetylcholine (ACh), respectively. Voltage-gated Ca channels are selective gates that regulate the majority of the Ca^2+^ influx into the cell [22], while nAChRs are non-selective cation channels that are permeable not only to Ca^2+^ but also to other cations [23]. AChRs can be classified into nicotinic and muscarinic AChRs. While nAChRs function as ionotropic receptors inducing as a fast response in the cell [24], mAChRs are coupled to G-proteins that use the transmitted signal to activate a cascade of reactions and produce a secondary messenger. Compared to nAChRs, mAChRs provide an indirect response to a series of reactions [25].

In this study, we investigated the effects of GYM A and SPX 1 on the intracellular Ca^2+^ ([Ca]_i_) alterations mediated by ion channels and receptors. We chose PC12 cells in order to be able to investigate the effects of toxins on more than one type of receptor and many different ion channels [20]. We specifically aimed to determine the influence of GYM A and SPX 1 on voltage-operated plasma-membrane Ca channels and nicotinic and muscarinic AChRs of PC 12 cells under physiological conditions. Since PC12 cells contain the nAChR subtypes α3, α5, β2–β4 [26], as well as atypical mAChR subtypes [27], our approach is more general than previously published works [14,15,16,17,18].

## 2. Results

### 2.1. Depolarization of Ca Channels Using K^+^

**Figure 2:**
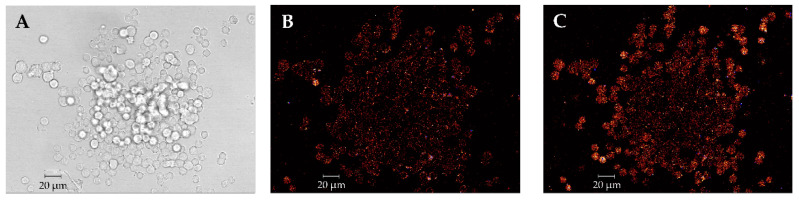
(**A**) Transmission image of pheochromocytoma (PC12) cells and (**B**) fluorescence images of flou-3 acetoxymethylester (Flou-3 AM)-stained PC 12 cells before and (**C**) during depolarization.

**Figure 3:**
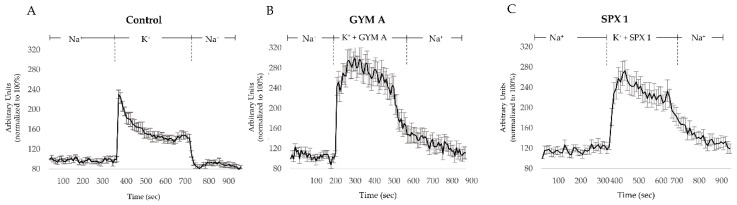
Alterations of fluorescence intensity (FI) induced by 80 mM K^+^. (**A**) Intracellular calcium levels ([Ca]_i_) changes (shown as arbitrary FI units) induced by activation of voltage-gated calcium channels (K^+^) under control conditions and (**B**) in cells treated with 0.5 µM GYM A or (**C**) 0.5 µM SPX 1. *n* = 10.

To account for the possibility of the toxins inhibiting voltage-dependent calcium channels, we investigated their influence on depolarization-induced [Ca]_i_ changes (Figure 2). This was done to avoid misinterpretation of toxin induced calcium channel inhibition with effects on AChRs. In the controls, an elevation of [Ca]_i_, induced by high concentrations of K^+^ was associated with a change in fluorescence emission intensity of the Flou-3 AM dye. Baseline recovery of [Ca]_i_ to the baseline was observed after depolarization (Figure 3A).

Cells incubated with GYM A or SPX 1 (0.5 µM) showed an increase of [Ca]_i_ during depolarization and no calcium channel inhibition (Figure 3B,C, respectively). In both treatments, the baseline recovery was delayed (Figure 3B,C) compared to the control (Figure 3A).

### 2.2. [Ca]_i_ Changes Induced by ACh

**Figure 4:**
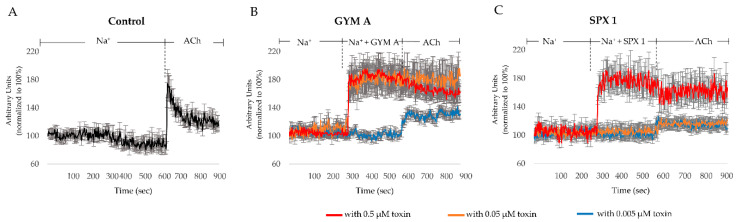
[Ca]_i_ changes induced by ACh, GYM A, and SPX 1. (**A**) [Ca]_i_ level changes induced by activation of AChRs under control conditions; (**B**) in cells treated with GYM A in the concentrations 0.005 µM (blue), 0.05 µM (orange), and 0.5 µM (red); and (**C**) SPX 1 in the concentrations 0.005 µM (blue), 0.05 µM (orange), and 0.5 µM (red). *n* = 10.

A [Ca]_i_ elevation, as described by an increase of [Ca]_i_ from the baseline, was also mediated by ACh (Figure 4A). To reveal the effects of the toxins on [Ca]_i_, GYM A or SPX 1 were applied before ACh (Figure 4B,C).

In cells treated with either 0.5 µM (Figure 4B, in red) or 0.05 µM (Figure 4B, in orange) GYM A, an increase of [Ca]_i_ was observed after the toxins were applied. The successive application of ACh induced no further [Ca]_i_ elevation (Figure 4B, in red and orange). A different pattern was observed for 0.005 µM GYM A (Figure 4B, in blue). In this case, no increase in [Ca]_i_ was observed after the addition of GYM A but instead after the ACh was applied.

The application of 0.5 µM of SPX 1 also resulted in an increase in [Ca]_i_ (Figure 4C, in red). Following the application of ACh, no further [Ca]_i_ response was detected. As for the lower concentrations, no increase in [Ca]_i_ was observed after the addition of 0.05 and 0.005 µM SPX 1. When ACh was subsequently applied, a [Ca]_i_ elevation was observed (Figure 4C, in orange and blue).

**Figure 5:**
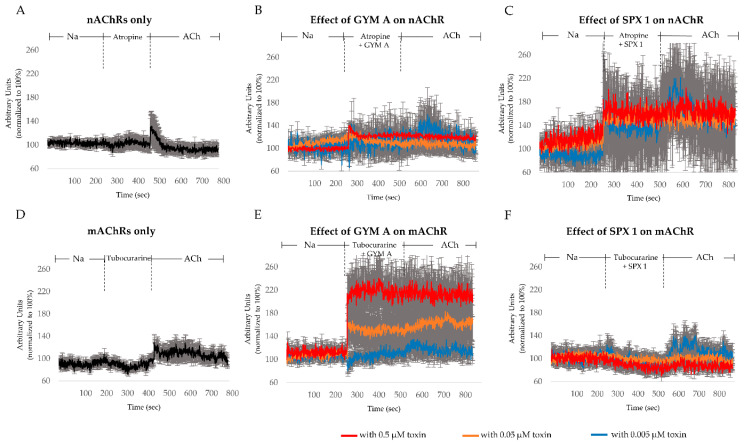
[Ca]_i_ changes induced by acetylcholine (ACh), GYM A, and SPX 1 through pharmacologically isolated nicotinic acetylcholine receptors (nAChRs) or muscarinic AChRs (mAChRs). (**A**) [Ca]_i_ level changes in cells pretreated with only mAChR-blocker atropine and (**B**) atropine with GYM A in the concentrations 0.005 µM (blue), 0.05 µM (orange), and 0.5 µM (red) followed by the application of ACh. (**C**) [Ca]_i_ level changes in cells pretreated with mAChR-blocker atropine and SPX 1 in the concentrations 0.005 µM (blue), 0.05 µM (orange), and 0.5 µM (red) followed by the application of ACh. (**D**) [Ca]_i_ level changes in cells pretreated with only nAChR-blocker tubocurarine and (**E**) tubocurarine with GYM A in the concentrations 0.005 µM (blue), 0.05 µM (orange), and 0.5 µM (red) followed by the application of ACh. (**F**) [Ca]_i_ level changes in cells pretreated with nAChR-blocker tubocurarine and SPX 1 A in the concentrations 0.005 µM (blue), 0.05 µM (orange), and 0.5 µM (red), followed by the application of ACh. *n* = 10.

To differentiate the effects of both toxins on AChRs present in the cells, we pharmacologically isolated the effect of the toxins on nAChRs by using the mAChR-blocker atropine (Figure 5A–C). Under control conditions, a response in [Ca]_i_ was observed upon the addition of ACh to nAChRs (Figure 5A). In cells treated with atropine and 0.5 µM GYM A (Figure 5B, in red) and 0.05 µM GYM A (Figure 5B, in orange), a [Ca]_i_ response was observed. The subsequent application of ACh induced no further increase in [Ca]_i_. For 0.005 µM GYM A (Figure 5B, in blue), only a slight increase of FI (~10%) was observed when the toxin was added along with atropine. Upon application of ACh (Figure 5B, in blue), [Ca]_i_ increased further (~30% FI increase). When SPX 1 (0.5, 0.05, and 0.005; Figure 5C, in red, orange, and blue, respectively) was applied together with atropine, an increase in [Ca]_i_ was observed at all three concentrations. Upon application of ACh, no additional [Ca]_i_ response was observed in 0.5 and 0.05 µM SPX 1-treated cells. However, in cells treated with 0.005 µM SPX 1 (Figure 5C, in blue), an increase in [Ca]_i_ by ACh was evident.

The effect of the toxins on mAChRs was investigated by adding nAChR-blocker tubocurarine. Under control conditions, a response in [Ca]_i_ was observed upon the addition of ACh to mAChRs (Figure 5D). In cells treated with 0.5, 0.05, and 0.005 µM GYM A, a dose-dependent increase of [Ca]_i_ was observed (Figure 5E, in red, orange, and blue, respectively). At the highest concentration used in this study (0.5 µM), no further [Ca]_i_ response was observed after additional ACh was applied (Figure 5E, in red). For the two lower concentrations, 0.05 and 0.005 µM, an increase of [Ca]_i_ (~20% FI increase) was observed (Figure 5E, in orange and blue, respectively). A different response was observed when SPX 1 was applied on mAChRs. A decrease of [Ca]_i_ (~5–10% FI) was measured in cells treated with SPX 1 (Figure 5F, in red, orange, and blue, respectively). In cells treated with 0.5 and 0.05 µM SPX 1 (Figure 5F, in red and orange, respectively), the addition of ACh resulted in no further change in [Ca]_i_. In cells treated with 0.005 µM SPX 1 (Figure 5F, in blue), however, the addition of ACh induced a [Ca]_i_ elevation (~+40% FI).

**Figure 6:**
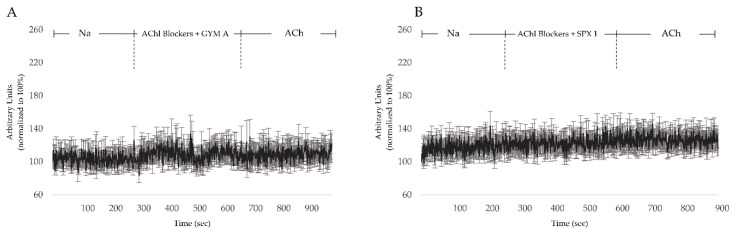
(**A**) [Ca]_i_ level changes in cells pretreated with mAChR-blocker atropine and nAChR-blocker tubocurarine with 0.5 µM GYM A and (**B**) SPX 1. *n* = 10.

In order to rule out effects of the toxins on cellular targets involved in [Ca]_i_ signaling other than AChRs, either GYM A or SPX 1 (0.5 µM) was applied to the cells while simultaneously inhibiting nAChRs and mAChRs. In both treatments, no obvious change in [Ca]_i_ was observed. In addition, no [Ca]_i_ response was observed after ACh addition (Figure 6A,B).

## 3. Discussion

### 3.1. GYM A and SPX 1 Do not Inhibit the Influx of [Ca]_i_ through Voltage-Gated Ca Channels

First, we excluded the influence of voltage-gated Ca channels on toxin-induced [Ca]_i_ changes. During depolarization, voltage-gated Ca channels mediate the influx of Ca^2+^ into the cell. Free Ca^2+^ is removed from the cytosol by uptake into the endoplasmic reticulum and mitochondria and by extrusion mechanisms such as calcium pumps and ion exchangers [28,29]. [Ca]_i_ measurements during depolarization in controls as well as in GYM A- and SPX 1-exposed cells indicate that the toxins do not reduce the influx of [Ca]_i_ through voltage-gated Ca channels. The gradual decrease in [Ca]_i_ following depolarization in GYM A- and SPX 1-treated cells (Figure 3B,C, respectively) indicates either an inhibition of transport proteins (e.g., Ca^2+^ ATPases) that remove Ca^2+^ from the cell or that the influx of Ca^2+^ into the cell is mediated by other means [28]. Since AChRs depolarize the cellular membrane and permeate Ca^2+^ into the cells [23], and GYM A and SPX 1 bind to those receptors, AChRs were chosen as targets for further experiments.

### 3.2. GYM A and SPX 1 Alter [Ca]_i_

The binding of an agonist such as ACh to AChRs initiate the influx of Ca^2+^ into the cell. GYM A or SPX 1 tested here had the same effect: the application of 0.05, 0.5 µM GYM A, and 0.5 µM SPX 1 induced [Ca]_i_ elevations (Figure 4B, in red and orange and Figure 4C, in red). The activation of AChRs by either GYM A or SPX 1, particularly at the concentration of 0.5 µM, may be the reason why a slow and gradual decrease in [Ca]_i_ was observed in the K^+^-depolarized cells (Figure 3B,C). The response observed in [Ca]_i_ infers that toxins mimic ACh and thereby initiate the influx of Ca^2+^ into the cell. The resulting AChR-toxin complex further inhibits a subsequent activation of AChRs by ACh (Figure 4B, in red and orange and Figure 4C, in red). Both toxins activated AChRs at varying concentrations, with GYM A acting as a more potent activator than SPX 1. Compared to GYM A (at 0.05 µM, Figure 4B, in orange), SPX 1 of a higher concentration (at 0.5 µM, Figure 4C in red) was needed to induce comparable Ca^2+^ influx into the cell. At the lowest concentrations (0.005 µM) of both toxins, no activation of AChRs was measurable.

### 3.3. GYM A and SPX 1 Show a Similar Effect on nAChRs and a Differential Response to mAChRs

The difference in response in [Ca]_i_ between the atropine-treated cells (Figure 5A) and atropine-toxin-treated cells indicate an interaction of GYM A (Figure 5B) and SPX 1 (Figure 5C) with nAChRs. Both toxins activate nAChRs, induce entry of Ca^2+^ into the cells, and block the binding of ACh to nAChRs. At low concentrations (0.005 µM), activation of nAChRs appears in SPX 1- (Figure 5C, in blue) but not in GYM A-treated cells (Figure 5B, in blue), demonstrating dose-dependent differences. For both toxins, low concentrations did not inhibit the activation of nAChRs by ACh, resulting in an increase in [Ca]_i_. Our results support previous studies that showed SPX to have a higher efficacy for nAChRs than GYM A (2–15 fold difference) [16,17].

The contrasting response between cells treated only with tubocurarine and cells that were additionally treated with GYM A or SPX 1 suggests that toxins may have also interacted with mAChRs. GYM A activates mAChRs dose-dependently. ACh only activates mAChRs at low concentrations of GYM A (0.005 µM) (Figure 5E, in blue) where an inhibition of a constitutive activity of receptors may have happened. For SPX 1, a decrease in [Ca]_i_ was observed that could have been caused by an inhibition of a constitutive activity of mAChRs [30]. Following the observed decrease in [Ca]_i_ upon addition of SPX 1, high and moderate concentrations of the toxin inhibit the subsequent activation of mAChRs by ACh (Figure 5F, in red and orange). A low concentration of SPX 1, on the other hand, had no effect (Figure 5F, in blue). GYM A [17] and SPX 1 [15,17] have previously been described to have a low ability to interact with mAChRs, with the latter having a lower affinity [17]. Here, we show a differential effect of GYM A and SPX 1 on mAChRs.

### 3.4. GYM A and SPX Alter [Ca]_i_ through nAChRs and mAChRs

At a concentration of 0.5 µM, GYM A and SPX 1 activate nAChR (GYM A also activates mAChRs) and inhibit the response to ACh. [Ca]_i_ was not affected by GYM A and SPX 1 (0.5 µM) when both AChR subtypes were blocked simultaneously by atropine and tubocurarine (Figure 6A,B). This shows that nAChRs and mAChRs must be the primary targets of GYM A and SPX 1 related to [Ca]_i_ signaling.

Previous studies have demonstrated that both GYM A and SPX 1 acted as antagonists to nAChRs [14,15,16,17,18], while neither interacted strongly with mAChRs [15,17]. Here, we show that GYM A and SPX 1 activate nAChR at 0.5 µM and dose-dependently interact with a successive ACh stimulation. We show that GYM A additionally activates mAChRs at 0.5 µM demonstrating that mAChRs are also a target of GYM A. This may be due to different receptor subtypes present in PC12 cells (nAChR subtypes α3, α5, β2–β4 [26], and atypical mAChR subtypes [27]) compared to subtypes used in studies published previously (nAChR subtypes α7, α1_2_β1γδ, α7-5HT_3_, α3β2 and α4β2, and M_1_–M_5_ mAChR subtypes) [14,15,16,17,18]. To investigate structure–activity relationship and to investigate functional measurements due to desensitization mechanism, future electrophysiological studies are required. The potentiation of nAChRs by atropine is described for specific subunits of the receptor (α4β4) [31], which are to our knowledge not present in PC12 cells. A potential interaction site of atropine with nAChRs (inhibition) is the α3β4 subunit, which is present in PC12 cells [32]. It is therefore possible that in our experiment atropine potentiated the effect of SPX 1 on certain nAChR subunits and has a small effect during application of a low dose of GYM A. This provides a baseline for future studies.

The response of cells following direct application of higher concentrations of toxins has not been described previously. This study demonstrates GYM A and SPX 1 to dose-dependently interact with AChRs of neuroendocrine PC12 cells. Furthermore, SPX 1 has the capability to inhibit the constitutive activity of mAChRs.

The activation of receptors at high toxin concentrations and inhibition of subsequent activation by ACh at lower concentrations hint at the cooperativity of molecules. In this case, e.g., one molecule binds to one receptor’s binding site, preventing further activation by ACh, and when more toxin molecules together bind to more receptor sites, this leads to activation of receptors. The cooperativity can be calculated by the steepness of dose response relationships. This is unfortunately not feasible for our data due to limited amounts of compounds being available and therefore limited dose-response-related data points.

In summary, we show that under physiological conditions, both toxins act as agonists for nAChRs and that GYM A induces an increase in [Ca]_i_ through mAChRs. We provide clear evidence that GYM A and SPX 1 mimic the action of ACh, preventing further activation of receptors. The macrocyclic nature of GYM A and SPX 1 allows the toxins to conform to the same binding sites of nAChRs with the cyclic imine as the pivot point of the molecule [16]. Absence of a triketal ring system may provide a basis for selective activation of mAChRs by GYM A as compared to SPX 1. The difference in activity regarding nAChRs and mAChRs observed between the toxins may be due to the subtype selectivity of these receptors. Subtypes of nAChRs, which can be homomeric or heteromeric in form, have different permeabilities to Ca^2+^ and affinities to ACh [33]. mAChR subtypes couple to different G-protein types, initiating different secondary induction pathways [34]. Structural conformation of the toxins (presence or absence of a triketal ring system) as well as of the AChR subunits plays crucial roles in the selectivity and specificity of toxin–receptor interactions.

## 4. Materials and Methods

### 4.1. Extraction and Purification of Gymnodimine A and 13-Desmethyl Spirolide C

GYM A and SPX 1 were isolated from clonal isolates of *Alexandrium ostenfeldii* (OKNL 48) collected from Ouwerkerkse Kreek, the Netherlands [5]. The extraction and purification of the toxins was conducted using the procedure described by Zurhelle et al. [9]. In brief, the microalgal culture was treated with acetone (7% final concentration) and the toxins were extracted using conditioned HP-20 (Diaon Supelco, Steinheim, Germany). The resin was eluted with methanol, and the eluate was dried under vacuo before loading into the preparative reversed phase chromatography (C18, 25 × 310 mm, 5 mL min^−1^). The elution was done with a stepwise gradient from aqueous:acetonitrile (ACN) (water/ACN, 80:20 *v*/*v*) to 100% ACN.

### 4.2. PC 12 Culture Methods

Prior to cell cultivation, cover slips were placed into the Petri dishes, coated with 0.5 mg mL^−1^ collagen A (Biochrom, Berlin, Germany), and dried for 24 h. The Petri dishes were filled with 100 mL of culture medium composed of Roswell Park Memorial Institute (RPMI) medium 1640, 10% fetal calf serum, 5% horse serum, and 100 units penicillin/streptomycin per milliliter. Rat pheochromocytoma (PC12) cells (ATCC, Wesel, Germany) were then seeded into thus-prepared Petri dishes. The cells were kept in an incubator at 37 °C, 90% humidity, and 5% CO_2,_ and medium changes were conducted after three to five days of cultivation.

### 4.3. Fluorimetric Measurements of Intracellular Calcium Levels

For fluorometric measurements of [Ca]_i_, cover slips adhered with PC12 cells were incubated in Na^+^ buffer (in mM: 125 NaCl, 2.5 KCl, 1 MgCl_2_, 2 CaCl_2_, 1.3 NaH_2_PO_4_, 30 Glucose, and 26 Na HEPES (4-(2-hydroxyethyl)-1-piperazineethanesulfonic acid)) with a final concentration of 10 μM Ca^2+^ fluorescent dye, Flou-3 acetoxymethylester (Flou-3 AM), for 1 h at 37 °C. Then, the physiological Na^+^ buffer was removed and replaced with fresh Na^+^ buffer. The cell fluorescence was monitored using an inverted confocal laser scanning microscope (Leica SP5, Wetzlar, Germany) equipped with an argon ion laser for fluorescence excitation (exc 488 nm, em 520–550). Laser settings were identical in all runs and images were taken every second. The beam of the laser scanned the object plane through a Zeiss (Jena, Germany) 20x water immersion objective. We constructed a plastic inlay to reduce the exchangeable volume to 250 µL, to increase the speed of solution exchange and to minimize the amount of toxins used in the experiment. To increase speed, the compounds were manually pipetted, instead of using pumping or gravity filtration. The experiment took place in an Utermöhl chamber.

10 PC12 cells (*n* = 10) were selected in the cover slips and were analyzed independently using region of interest (ROI)s in the Leica Application Suite Advanced Fluorescence (LAS AF, Wetzlar, Germany) software. The dye intensity represents the calcium concentration inside the cell. Ten individual cells were measured simultaneously in all treatments. The cellular fluorescence emission of the selected PC12 cells was normalized by dividing the fluorescence measured by the initial values (t0) and then multiplying them by 100. All experiments were replicated using a different cell culture batch obtaining comparable results.

### 4.4. [Ca]_i_ Measurements Using K^+^ Depolarization

Cover slips with adhered cells were mounted on the microscope using a chamber with a volume capacity of 250 µL. The fluorescence of the PC12 cells was measured, and cells were subsequently depolarized by submerging K^+^ buffer (in mM: 55 NaCl, 80 KCl, 1 MgCl_2_, 2 CaCl_2_, 1.3 NaH_2_PO_4_, 30 Glucose, 26 Na HEPES) through manual pipetting. The effects of the toxin were determined by spiking the K^+^ buffer with GYM A or SPX 1 reaching a final concentration of 0.5 µM.

### 4.5. [Ca]_i_ Measurements with Application of Acetylcholine

Cells were stimulated by application of 100 µM Acetylcholine-Cl (ACh) (Sigma, Darmstadt, Germany) in Na^+^ buffer (described in Section 4.3). The effects of the toxin were determined by spiking the buffer with either GYM A or SPX 1 resulting in final concentrations of 0.005, 0.05, and 0.5 µM. In addition, AChR subtype blockers, atropine and turbocurarine, were utilized in order to investigate the mechanism of action against nAChR and mAChR, respectively. Both substances were used at a final concentration of 100 µM.
